# The Tumor Microenvironment of Epithelial Ovarian Cancer and Its Influence on Response to Immunotherapy

**DOI:** 10.3390/cancers10080242

**Published:** 2018-07-24

**Authors:** Galaxia M. Rodriguez, Kristianne J. C. Galpin, Curtis W. McCloskey, Barbara C. Vanderhyden

**Affiliations:** 1 Cancer Therapeutics Program, Ottawa Hospital Research Institute, 501 Smyth Road, Ottawa, ON K1H 8L6, Canada; garodriguez@toh.ca (G.M.R.); kgalpin@ohri.ca (K.J.C.G.); cmccloskey@ohri.ca (C.W.M.); 2 Department of Cellular and Molecular Medicine, University of Ottawa, 451 Smyth Road, Ottawa, ON K1H 8M5, Canada

**Keywords:** epithelial ovarian cancer, tumor microenvironment, tumor infiltrating lymphocytes, tumor-associated antigens, ascites, immunosuppression, prognostic factors, cancer-associated fibroblasts, exosomes, adipocytes

## Abstract

Immunotherapy as a treatment for cancer is a growing field of endeavor but reports of success have been limited for epithelial ovarian cancer. Overcoming the challenges to developing more effective therapeutic approaches lies in a better understanding of the factors in cancer cells and the surrounding tumor microenvironment that limit response to immunotherapies. This article provides an overview of some ovarian cancer cell features such as tumor-associated antigens, ovarian cancer-derived exosomes, tumor mutational burden and overexpression of immunoinhibitory molecules. Moreover, we describe relevant cell types found in epithelial ovarian tumors including immune cells (T and B lymphocytes, Tregs, NK cells, TAMs, MDSCs) and other components found in the tumor microenvironment including fibroblasts and the adipocytes in the omentum. We focus on how those components may influence responses to standard treatments or immunotherapies.

## 1. Introduction

An increasing body of evidence strongly suggests that the immune system is able to identify, control and eliminate nascent neoplastic cells in a process known as cancer immunosurveillance [1]. Epithelial ovarian cancers (EOCs) are “immunogenic tumors” that produce spontaneous antitumor immune responses detectable in peripheral blood, tumors and ascites of patients [2,3,4]. The resulting presence of tumor infiltrating lymphocytes (TILs) is associated with improved survival in EOC [5]. Unfortunately, there are a number of factors in the tumor microenvironment (TME) that can impair the presence or activity of TILs, thereby facilitating cancer progression.

Various immunotherapeutic strategies are attempting to address the challenges posed by the highly immunosuppressive EOC TME. Immunotherapies encompass many modalities, including immune checkpoint blockade, antibody-based therapies, cancer vaccines, cytokines, adoptive cell transfer, and chimeric antigen receptor-modified T cells [6]. However, emerging cancer immunotherapies (blocking antibodies for checkpoint inhibitors) have shown low rates of responses in EOC (reviewed in [2]). Improving this response rate is a major goal, which can only be achieved with a better understanding of the elements in the TME that contribute to treatment failure. Immune cells are the main players in the development of antitumor immunity or tumor progression, but there are also other components in the TME that should be taken into consideration when designing new therapeutic strategies. Those components include EOC-derived exosomes, cancer-associated fibroblasts (CAFs) and adipocytes residing in the omentum.

In this review we will describe those elements of the TME, how they influence the burden of the tumor, the responses to therapies, and their relevance in designing cancer immunotherapies for EOC.

## 2. Cancer Cells and Tumor Antigens

The success of cancer immunotherapy hinges on the ability to generate cancer-specific antitumor T-cell responses, to both recognize tumor-associated antigens (TAAs) and kill tumor cells, and to generate memory responses. TAAs can be classified into different categories: tissue differentiation, cancer testes antigens (CTAs), neoantigens derived from mutations, overexpressed cellular, splice variant, glycolipid, and viral antigens [7,8]. Ideal TAAs for immunotherapy targets are immunogenic and are expressed or overexpressed in tumor tissue, with restricted expression in associated normal tissues, in a significant percentage of patients [9]. Positive responses to immunotherapies such as immune checkpoint inhibitors [blocking programmed cell death protein 1 (PD-l), programmed death-ligand 1 (PD-L1) and cytotoxic T-lymphocyte-associated antigen 4 (CTLA-4)], have been associated with high mutation/neoantigen burden [10,11]. The initial clinical studies of small numbers of EOC patients treated with immune checkpoint inhibitors have resulted in clinical benefits in less than 20% of patients (Table 1). Unfortunately, little is known about the TME at the start of treatment in most studies, making it impossible to discern the factors that may have blocked any response. The failure to respond could be related to the neoantigen burden in EOC, which may be insufficient to generate a significant antitumoral response [12,13]. There are currently intense research efforts to understand other TAAs (Table 2) recognized by TILs to design informed immunotherapy targets (Table 3).

### 2.1. Neoantigens

Ovarian cancer has been shown to harbor an intermediate neoantigen load by whole exome sequencing/next generation sequencing [12,59]. Whole exome sequencing of tumor cells from ascites samples of three high-grade serous ovarian cancer (HGSC) patients revealed a tumor mutation burden (TMB) of approximately 20–40 mutations across all patients, however only 1/79 mutations (1.3%) were recognized by autologous tumor-associated T cells [60]. Comprehensive genomic profiling of ovarian cancer revealed low overall TMB among subtypes: HGSC (3.6), low-grade serous (LGSOC) (2.7), endometrioid (2.7), mucinous (2.7), and clear cell (2.7). Only a small percentage of patients had a significant TMB (20 or more mutations per Mb), meaning only a small percentage of patients would be predicted to show favorable response to immune therapy [12]. Consequently, in clinical trials of checkpoint inhibitors in EOC, CTLA-4 inhibitors (Ipilimumab), PD1 inhibitors (Nivolumab and Pembrolizumab), and PD-L1 inhibitors (MS-936559 and Avelumab) had response rates of 5–20% [14,20,61] (Table 1). A notable exception is the highly aggressive small cell carcinoma of the ovary, hypercalcemic type which, despite being a monogenic cancer, has responsiveness to anti-PD1 immunotherapy [62].

Neoantigen depletion [63], intratumoral heterogeneity, and clonal evolution of primary tumors and metastases may influence immunosurveillance and response to immunotherapy [64,65]. Epithelial T-cell rich tumors show the lowest amount of clonal diversity, neoantigen diversity and greatest loss of human leukocyte antigen (HLA) expression, which suggests immunoediting in the TME. T-cell poor tumors or “cold tumors” have a higher predicted and more diverse neoantigen load (unedited) [63].

### 2.2. Cancer Testes Antigens

CTAs are encoded by ~140 genes that are normally only expressed in germ cells (testes, placenta, fetal ovary) and not normal somatic adult cells, but often highly expressed in tumors. This along with their immunogenicity makes them significant targets for cancer immunotherapy [9,66,67]. Vaccination with recombinant MAGE-A3 antigen has been used in Phase I/II clinical trials for melanoma [68] and non-small-cell lung cancer (NSCLC) [69] with a good safety profile and observed humoral response, but only slight effects on survival. 

Several CTAs have been described in EOC (Table 2) and have been proposed as immunotherapy targets (Table 3) based on their tissue specificity and high expression in a significant number of EOCs of all subtypes. NY-ESO-1 (ESO_157–165_) specific CD8+ T cells were found in TILs of 71% of (10/14) vaccination naïve seropositive patients, and ex vivo proliferation of NY-ESO-1 specific peripheral blood lymphocytes in 65% of patients suggested that an adaptive immune response against this CTA can be achieved [70,71]. Clinical trials have subsequently tested the feasibility of generating NY-ESO-1 specific immune responses (Table 3). These approaches have generated humoral and CD4+ and CD8+ antigen specific T-cell responses, and in some cases, long lasting/complete responses [44,45,46,47]. NY-ESO-1 was not expressed in some recurrent tumors, raising the possibility of immune escape [44]. Furthermore, NY-ESO-1 reactive CD8+ T cells often express higher levels of inhibitory molecules lymphocyte-activation gene 3 (LAG3), PD-1 and CTLA-4, suggesting immunosuppression as a reason for lack of complete response during clinical trials [71].

Many characteristics of CTA epitopes and all TAAs such as (i) immunogenicity; (ii) restriction to HLA-I or -II; (iii) natural processing; (iv) expression; and (v) role in tumor progression remain to be elucidated and require validation in larger sample sizes. While the expression of CTAs does not often correlate with improved survival, their tissue specificity makes CTAs attractive targets for immunotherapies (Table 3) such as peptide vaccines [44,70], antigen-loaded dendritic cell (**DC**) vaccines [72], or oncolytic viral platforms, and for combined interventions with immune checkpoint inhibitors [73] or chemotherapy [74], in order to overcome tumor escape mechanisms.

### 2.3. Other TAAs

Genetic and epigenetic aberrations in cancer cells, resulting from mutations, amplifications or deletions in genes, provide both therapeutic targets and potential TAAs for immunotherapy design (Table 2). However, the greatest hurdles still remain in designing immunotherapeutic targets for a disease in which such aberrations, with the exception of p53 mutation (95% of HGSC [33,75]), are relatively uncommon (<20% frequency in HGSC cases) and lack antigen specificity to the tumor. Immunogenic oncogenes p53, Her2-neu and WT1 are broadly overexpressed in EOC, particularly HGSC, and targeted immunotherapies have been explored in clinical trials (Table 3). Other common but infrequent amplifications, mutations or deletions occur in *CCNE1*, *NF1*, *PTEN*, *KRAS*, *RB*, *CDK2NA*, *PIK3CA* and *AKT1/2* and provide potential therapeutic targets for EOC immunotherapy [33]. The DCs, T-cells, and peptide-based vaccine strategies against proteins described above have largely demonstrated immunological responses including CD4+ and CD8+ T-cell responses in preliminary clinical trials following vaccination, but often in the absence of clinical responses. This is perhaps due to widespread immunosuppression in the TME preventing T-cell activation and proliferation, as well as tumor heterogeneity and immunogenicity that impede proper TAA presentation to the immune cells.

The EOC immunopeptidome was profiled by isolating HLA molecules primarily from HGSC tumors and which were analyzed by mass spectrometry [57]. The analysis identified relevant proteins including CRABP1/2, FOLR1, and KLK10 presented on major histocompatibility complex (MHC) I molecules, and mesothelin, PTPRS and UBB presented on MHC-II molecules [57]. The most abundantly detected protein presented on MHC-I molecules was MUC16 (CA-125), with 113 different peptides expressed in approximately 80% of patients. MUC16-derived peptides were highly immunogenic (85% T-cell responses in vitro), and consequently it was proposed as the top candidate for targeted immunotherapy moving forward [57]. Although CA-125 is immunogenic, the large number of trials with a monoclonal antibody targeting CA-125 (Table 3) have been mostly unsuccessful as a monotherapy [76]. This failure could be explained by the weak magnitude of the immune response generated, the loss of expression or down-regulation of CA-125 on EOC cells to avoid immune recognition, or the overgrowth of CA-125(-) EOC cells as a consequence of cancer immunoediting process.

A single TAA is generally only expressed in a subset of patients, making the design of a universal immunotherapy challenging. The main barrier of targeting a single TAA is cancer immunoediting, which enables the enrichment of neoplastic cells in tumors that do not express the targeted TAA over time. Chimeric antigen receptor T (CAR-T) cells provides the option of combining multiple antigen specificities, and delivering direct cytokine stimulation (GM-CSF, IL-12) to the TME, irrespective of the MHC status of the patient [8].

### 2.4. Tumor Immunogenicity and Other Immunoinhibitory Molecules

Loss of immunogenicity is an immune hallmark of cancer that is exploited by tumors to evade immune recognition. This can be triggered by down-regulation or loss of expression of MHC-I and -II, and the antigen processing and presentation machinery (APM) [77,78,79,80]. Expression of MHC-I genes is altered by 60–90%, depending on the cancer type. These impairments reduce the antigens presented on the cell surface leading to decreased or lack of recognition and elimination by cytotoxic T lymphocytes.

The mechanisms that are related to immune cell infiltration in EOC are dependent on MHC-I and -II status [3,81]. The presence of neoantigen-reactive T cells in patients with EOC can improve survival [82]. However, as mentioned before, since ovarian tumors possess intermediate/low mutation burdens, the incidence of naturally processed and presented neoantigens generating a significant antitumoral response is very low [13]. The expression of APM components and the presence of intratumoral T-cell infiltrates were significantly associated with improved survival [81]. Han. et al. demonstrated that the majority of ovarian carcinomas analyzed had either heterogeneous or positive expression of peptide transporter 1 (TAP1), TAP2, HLA class I heavy chain, and beta-2 microglobulin [81]. Concurrent expression of HLA-DR and CA-125 on cancer cells correlated with higher frequency of CD8+ TILs and increased survival [83]. Similarly, tumor cell expression of HLA-DMB was associated with increased numbers of CD8+ TILs and both were associated with improved survival in advanced-stage serous EOC [84]. The regulation of APM components and MHC molecules in human cancers is a significant area of research but is beyond the scope of this review (reviewed in [85,86]).

The mutational profile of EOC can also predict immunogenicity. Tumors with deficient homologous recombination (HR) machinery occur with a frequency of up to 50% [33]. These include mutations in *BRCA1/BRCA2* (20% frequency) or non-BRCA HR deficiencies (Fanconi anemia genes, restriction site associated DNA genes, and DNA damage response genes) [33]. HR deficient tumors have higher predicted neoantigen load, and infiltrating and peritumoral lymphocytes in these tumors have increased PD-1/PD-L1 expression [43], which may enhance susceptibility to immune checkpoint therapy. *BRCA1/2* mutated HGSC tumors have more CD3+ and CD8+ TILs compared to HR-proficient tumors, a signature associated with higher overall survival [43,87]. p53 mutations are also associated with higher levels of TILs [87,88]. Non-HR deficient tumors therefore have poorer overall survival [43] and may be less immunogenic, making them more difficult to target with immunotherapies. Alternative strategies and TAAs to target this group of EOC tumors need further investigation.

The expression of immunoinhibitory molecules on cancer cells, including PD-L1 and Indoleamine 2,3-dioxygenase (IDO) are associated with patient prognosis. Higher expression of PD-L1 on tumor cells correlates with poorer prognosis, suggesting that the PD-1/PD-L pathway can be a good target for restoring antitumor immunity in EOC [89,90], although others have suggested that high PD-1/PD-L1 expression in primary tumors may be associated with a favorable progression-free survival [91,92]. Increased infiltration of CD8+ T cells is associated with high PD-L1 expression likely as a result of an adaptive response where infiltrating CD8+ T cells secrete interferon gamma (IFNγ) that subsequently induces PD-L1 expression on cancer cells. This in turn inhibits T-cell activation and proliferation, preventing successful targeting and clearance of the tumor. Immune checkpoint inhibitors (anti-PD-L1 and PD-1) have been FDA approved for melanoma and NSCLC, but only a small percentage (10–33%) of ovarian cancers express PD-L1 [61,92,93], thus only a small percentage of patients may respond to anti-PD-L1 immunotherapy (Table 1). The enzyme IDO is often overexpressed by cancer cells, but is also produced by DCs and macrophages [94,95] in the TME. IDO catabolizes tryptophan, which leads to cell cycle arrest or apoptosis in NK and CD4 T cells [96], and skewed differentiation of regulatory T cells (Tregs) induced by plasmacytoid DCs, leading to immunosuppression in the TME [97]. Positive staining for IDO, observed in 24–57% of patient samples, is associated with poor prognosis of HGSC, decreased CD8+ TILs, as well as resistance to chemotherapy [98,99]. Targeting IDO with inhibitors may improve outcome [100,101].

## 3. Immune Cells

Most solid tumors are infiltrated by myeloid- and lymphoid lineage-derived immune cells that are differentially distributed within the TME with a crucial role in the establishment of antitumoral responses or tumor progression [1]. Growing tumor cells release “danger signals” that enable the recruitment of immune cells into the tumor niche. TILs such as CD4+ and CD8+ T cells, B lymphocytes, Natural Killer (NK)-T cells, as well as innate immune cells such as NK cells, macrophages and DCs, are then recruited in order to eliminate nascent neoplastic cells, acting as an extrinsic tumor suppression mechanism [102]. However, immunosurveillance promotes the selection of poorly immunogenic cancer cells through cancer immunoediting where neoplastic cells that resist the elimination phase can persist in equilibrium with effector CD4+ and CD8+ T cells under a pro-inflammatory milieu. Over time, cancer cells with the most immunoevasive characteristics are selected, enabling them to eventually escape immune attack [102]. Finally, immunoedited tumors become clinically apparent with variants that trigger the establishment of an immunosuppressive TME containing immunosuppressive immune cells such as myeloid-derived suppressor cells (MDSCs), Tregs, and others [2,103].

### 3.1. Immune Modulators and Adaptive Immune Cells in the Ovarian Cancer TME

#### 3.1.1. TILs

**Figure 1:**
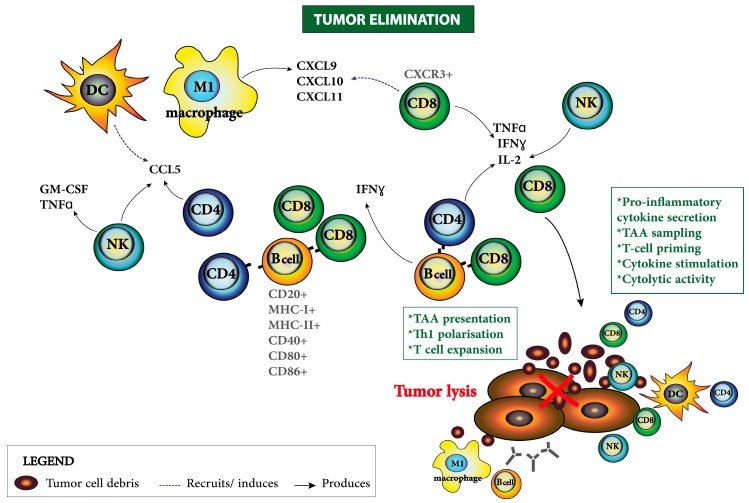
Antitumoral responses in the EOC TME. Immunogenic cell death induces the release of DAMPs mediating the recruitment of innate cells and APCs. Lympho-attracting chemokines produced by APCs such as macrophages enable the recruitment of CD8+ T cells to the tumor niche. DCs are also attracted by the production of CCL5 derived from NK cells and CD4+ T cells. The pro-inflammatory milieu enables TAA sampling and presentation by APCs to T cells to induce their activation and expansion. Pro-inflammatory cytokines released by activated effector T cells, M1 macrophages and DCs allow the amplification of the antitumoral response, enabling the cytolytic death of EOC targeted by CD8+ TILs and NK cells. B cells also participate in antitumor immunity by presenting TAAs to CD8+ T cells, by facilitating Th1 polarization, T-cell expansion and by producing tumor specific antibodies. Danger-associated molecular patterns (DAMPs), Antigen presenting cells (APCs), tumor-associated antigens (TAAs), dendritic cells (DCs), natural killer cells (NKs), CD4+ T helper cell (Th1).

TILs can localize into the tumor islet (intraepithelial) and in the peritumoral space (stromal) [2]. Several studies have shown a positive correlation between the presence of intraepithelial TILs and tumor regression in many solid cancers [4,5,104,105,106,107]. T cells can be found in primary tumor tissue and omental metastases [4,104,105,107,108,109,110,111] and their presence has been correlated with positive prognosis. Dadmarz et al. demonstrated that TILs isolated from EOC patients (primary tumor, metastases or ascites) were tumor-specific and could recognize autologous TAAs. Antitumoral responses were mainly characterized by the secretion of tumor necrosis factor-alpha (TNFα) and granulocyte macrophage-colony stimulating factor (GM-CSF) when stimulated with autologous tumor [112]. Later, Zhang and colleagues showed that intraepithelial CD3+ TILs can be found in >50% of advanced-stage EOC with their presence correlating with a five-year overall survival rate of 38% in contrast to 4.5% in patients whose tumors contained no T cells [5]. Even after debulking and platinum-based chemotherapy, the presence of intraepithelial CD3+ TILs increased the five-year overall survival rate (>70%) in comparison to patients whose tumors contained no T cells in islets (11%) [5]. T cell-rich tumors correlated with delayed recurrence or death and were associated with increased expression of Interleukin-2 (IL-2), IFNγ and lymphocyte-attracting chemokines within the tumor such as CXCL9 [113], CCL21, and CCL22 [5]. Conversely, tumors with no T cells in islets were associated with an increased level of vascular endothelial growth factor (VEGF), an angiogenic regulatory factor in the TME associated with early recurrence and short survival [5]. A more recent study showed that intratumoral accumulation of CXCR3 ligands such as CXCL9 and CXCL10, predicts survival in advanced HGSC [113] (Figure 1). This study also identified the cyclooxygenase (**COX**) metabolite Prostaglandin E2 as a negative regulator of chemokine secretion that contributes to tumor progression by impeding TILs recruitment in ovarian cancer [113]. Further investigation showed that expression of both COX-1 and COX-2 were negatively correlated with intraepithelial CD8+ TILs as well as with EOC patient survival [114].

While some studies have reported that the presence of both intraepithelial CD3+ and CD8+ T-cells correlates with improved disease-specific survival for EOC patients [81,87] others have shown that this beneficial characteristic is attributed to intraepithelial CD8+ TILs [4,104,105,107,108,109,110,115]. No association was found for CD3+ TILs or other subtypes of intraepithelial or stromal TILs in EOC overall patient survival. Interestingly, the subgroups displaying high versus low intraepithelial CD8+/CD4+ TIL ratios had favorable survival prognosis (median = 58 versus 23 months) [106]. This was due to the unfavorable effect of CD4+ CD25+ forkhead box P3+ (FOXP3) Tregs [88,104,106] that will be discussed later.

In 2012, a meta-analysis of ten studies with 1815 ovarian cancer patients confirmed the prognostic value of intraepithelial CD8+ TILs in EOC specimens regardless of the tumor grade, stage, or histologic subtype studied [111]. Their presence suggests that spontaneously activated antitumoral responses are present in the tumor niche to control tumor outgrowth [111] as observed by the presence of tumor-reactive antibodies and T cells found in the peripheral blood of advanced stage EOC patients [116,117,118], and oligoclonal tumor-reactive T cells isolated from blood, ascites or tumors [88,119,120,121,122,123]. Conversely, the lack of intraepithelial TILs is significantly associated with poor survival among EOC patients [111]. Thus, immunotherapies aiming to increase the effector functions of pre-existing antitumoral CD8+ TILs and triggering effector T cell-trafficking to the TME are the holy grail of cancer immunotherapy.

CD4+ T cells as well as CD8+ T cells can specifically recognize TAAs from malignant cells. CD4+ T helper (Th) cells provide cytokine support for CD8+ T-cell proliferation and expansion to eliminate cancer cells and trigger antitumoral responses. In an analysis of ovarian tumors, Tsiatas et al. found that a high percentage of CD4+ CD25hi cells and activated CD4+ T cells were significantly associated with improved median overall survival [124]. Two other studies also showed a positive correlation of the high frequency of CD4+ TILs and EOC patient survival [110,125]. Nesbeth et al., using an animal model for EOC, found that tumor-primed CD4+ T cells produce high levels of CCL5 that enables the recruitment and activation of DCs to the TME. Mature DCs were then able to prime tumor-specific CD8+ T cells and confer long-term protection [126]. Hence, immunotherapies stimulating both effector CD4+ and CD8+ T cells could confer synergistic antitumoral responses.

#### 3.1.2. Regulatory T lymphocytes

Tregs negatively regulate antitumoral responses in both a direct and indirect manner, highlighting that Tregs are a fundamental means of tumor immune evasion [127,128]. In healthy tissues, Tregs mediate tolerance by suppressing autoreactive T cells to protect and prevent excessive tissue destruction. Since most TAAs are composed by self-peptides, Tregs are often found in tumors to dampen antitumoral responses. Tregs accumulate and are more frequently present in tumors, with a shift in the median ratio of Tregs to TILs from 3–8% in healthy tissue to 18–25% in all analyzed cancers, including EOC [129]. Curiel et al. analyzed 104 EOC specimens and found that CD4+ CD25+ FOXP3+ Tregs specifically suppress antitumoral T cells in vivo, contributing to tumor growth. In addition, their presence correlates with poor patient outcome [130]. CD4+ Tregs preferentially migrated to tumor and ascites and were rarely found in draining lymph nodes at later cancer stages [131]. Immunotherapies impeding Treg trafficking could release the TME immunosuppression and promote the development of antitumoral responses.

**Figure 2:**
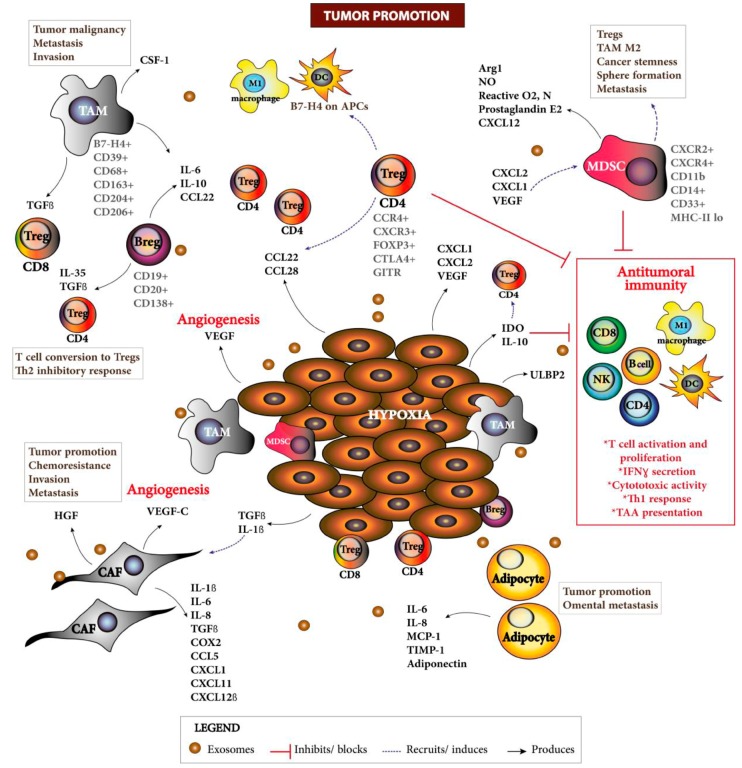
Tumor promoting network in the EOC TME. Outgrowth of EOC provokes hypoxia that induces the expression of chemokines to recruit MDSCs, Tregs, and TAMs. Tregs induce B7-H4 expression on APCs, subsequently blocking cytokine secretion, cytolytic activity, T-cell proliferation and promoting an immunosuppressive TME. EOC cells and MDSCs produce IDO that catabolizes tryptophan, rendering T cells anergic and dysfunctional. MDSCs and TAMs contribute to tumor growth, malignancy, metastasis and stemness. Several tumor promoting cytokines such as IL-6, IL-10 and TGFβ are prominent in the TME. VEGF released by EOC cells and CAFs stimulates angiogenic factors in the TME. CAFs also secrete many factors that mediate tumor cell migration, proliferation, invasion and chemoresistance, and contribute to the immunosuppressive TME. Adipocytes produce FA and cytokines that fuel tumor growth and omental metastasis. Myeloid-derived suppressor cells (MDSCs), tumor-associated macrophages (TAMs), regulatory T cells (Tregs), cancer-associated fibroblasts (CAFs), fatty acids (FA).

FOXP3+ Tregs express minimal levels of effector cytokines and granzyme B, but are able to induce inhibitory activities through IL-10 and transforming growth factor beta (TGF-β) production [132] and cell–cell interactions [127] (Figure 2). Barnett et al. showed that EOC tumors highly infiltrated by Tregs were associated with poor survival, advanced stage and suboptimal debulking [109]. Investigation of the influence of cytoreduction on the immune system of primary and recurrent EOC found that the ratio of CD4/CD8 is increased in primary but not in recurrent tumors [133]. Primary cytoreduction increased circulating effector CD4+ and CD8+ T cells, but circulating CD4+ Tregs were decreased as well as IL-10 serum levels, but not TGFβ and IL-6 [133]. CD4+ Tregs were also decreased after chemical debulking in patients treated with neoadjuvant chemotherapy. The reduction of the systemic and TME immunosuppression triggered by surgical debulking resulted in an increased capacity of CD8+ T cells to respond to the recall antigens, but not in patients who were previously subjected to chemotherapy or affected by recurrent EOC [133].

Fialová and colleagues studied the dynamics of the tumor-infiltrating immune cells during different stages of EOC [134]. Early stage disease displayed a strong Th17 immune response while stage II patients had responses characterized by the recruitment of Th1 cells. Disseminated disease (stages III and IV) were characterized by high amounts of Tregs, tumor-associated macrophages (TAMs), DCs, and high levels of CCL22, which is secreted by tumor cells, TAMs and DCs to enable further recruitment of Tregs and immunosuppression [134]. Other studies have shown the importance of the TME in facilitating the establishment of tolerance and recruitment of Tregs to sustain tumor growth. Using EOC cell lines in vitro, Facciabene et al. found that tumor hypoxia induces the expression of chemokine ligands such as CCL28, enabling the recruitment of Tregs and triggering angiogenesis [135]. CCL28 overexpression was associated with a poor outcome in patients with EOC [135]. Similarly, CCL22 production by TAMs enabled the recruitment of Tregs [130,134] that induced B7-H4 on antigen-presenting cells including macrophages [136]. CXCR3+ Tregs, able to control type-I T-cell responses, are highly enriched in EOC and represent the majority of Tregs [137]. These Tregs were able to suppress T-cell proliferation and IFNγ secretion [137].

An interesting study analyzed 22 EOC ascites specimens and found significantly elevated levels of IL-6, IL-8, IL-10, IL-15, IP-10, MCP-1, MIP-1β and VEGF and significantly reduced levels of IL-2, IL-5, IL-7, IL-17, PDGF-BB, and CCL5 compared to plasma. Moreover, T cells derived from EOC-associated ascites displayed poor responsiveness when expanded in vitro [138]. The authors claimed that this non-responsiveness could be explained by a high CD4/CD8 ratio that may indicate the presence of Tregs, reduced IL-2 and elevated IL-6 and IL-10 levels triggering a Th2 inhibitory immune response [138]. This high CD4/CD8 ratio was also associated with poor outcome [109,115,136,139], consistent with other studies [124,140,141]. In contrast, a positive correlation between Tregs and patient prognosis has been reported [140]. High tumor grade correlated with higher frequencies of CD3+, CD68+ CD163+ TAMs, and CD25+ FOXP3+ Treg cells, but Treg frequencies were significant predictors of favorable prognosis in patients with familial ovarian cancer (11/73 patients with *BRCA* mutation) [140]. The presence of FOXP3+ TILs may be linked to positive prognostic factors in optimally debulked HGSC patients [141]. Nevertheless, this disease-specific survival was positively associated along with other TIL markers such as CD8, CD3, TIA-1, CD20 (a B cell surface marker), MHC class I and class II [141].

CD8+ Tregs are also found in EOC [142,143]. They regulate the immunosuppressive TME by limiting immunosurveillance mechanisms and contributing to cancer progression [144]. Recently, Zhang and colleagues showed that CD8+ Tregs are found in the stroma and intraepithelial areas of EOC tumors [143]. CD8+ Tregs are characterized by the expression of FOXP3, CTLA-4, and CD25, but decreased expression of CD28 [143]. CD8+ Tregs were able to convert effector CD8+ T cells into suppressor cells [143]. CD8+ Tregs exert their suppressive function through the secretion of TGF-β1 [142].

Overall, Tregs are considered a critical barrier against antitumoral responses along with tolerance-inducing plasmacytoid DCs, B7-H4+ TAMs, MDSCs, IL-10, TGFβ, and VEGF. All these processes act in concert as a tumor evasion mechanism resulting in tumor progression [145]. Barriers to antitumoral responses are summarized in Figure 2.

#### 3.1.3. B Lymphocytes

B lymphocytes have been reported to have pivotal roles in cancer immunity [146]. Stromal or intraepithelial B lymphocytes have been found in EOC [141]; however their function in tumor development is not yet clear. Their presence is proposed to be associated with a good prognosis depending on the tumor stage and the TME where they are found [4,108,141]. The presence of B cells and CD8+ TILs correlates with increased patient survival compared to CD8+ TILs alone [108]. Nielsen et al. analyzed tumor and serum specimens obtained from patients with HGSC and found that the majority of CD20+ TILs were antigen experienced and suggested to accomplish TAA presentation in the TME since they often co-localized with CD8+ TILs and expressed markers such as MHC-I, MHC-II, CD40, CD80, and CD86 [108]. B cells can achieve antitumor immunity by secreting IFNγ, facilitating CD4+ Th cells to polarize to Th1 responses, and promote T-cell expansion by presenting TAAs [146]. Recently, the positive role of B cells among TILs at metastatic sites from patients with HGSC was reported [147]. B cells were often found in the stroma of metastases and were characterized by a strong memory response against TAAs by production of tumor-specific IgGs (Figure 1). Interestingly, these responses were amplified by chemotherapy [147].

Conversely, a new subset of B cells, regulatory B cells (Bregs), has been recently designated as immunosuppressive cells able to secrete anti-inflammatory mediators such as IL-10, IL-35, and TGF-β, triggering T-cell conversion to Tregs [148] (Figure 2). Indeed, a study that analyzed EOC tumor tissue and omental metastases found that high B cell infiltration negatively correlates with patient survival [149]. High CD20 and CD138 expression correlated with high tumor grade [149]. Analysis of omental specimens from patients with HGSC found that overall survival was 160.6 months in patients with low B-cell expression vs. 47.3 months in those with high B-cell expression, associating increased B-cell infiltration with poorer survival [150]. Similarly, the analysis of post-chemotherapy effusions from ovarian carcinomas revealed that a higher percentage of CD19+ cells (B cell marker) and stage IV disease predicted poor survival for patients [151].

Taken together, it is important to consider that several B-cell subsets with different phenotypes and functions exist, and they may have various roles in modifying the ability of tumors to respond to treatment [146]. Thus, a deep characterization of B-cell subpopulations within the TILs, ascites, and peripheral blood at different stages is crucial in order to provide a better understanding of the capability, importance and therapeutic potential of these cells in EOC.

#### 3.1.4. NK-T Lymphocytes

NK-T cells possess dual-functional capability: as T-cell subsets with a T-cell receptor (TCR)-mediated specific cytotoxicity and as NK cells with acquired killer functions [152,153]. NK-T cells have been found in increased frequencies in EOC tumor ascites compared to blood, but they were decreased at higher tumor grade and in cases of platinum resistance [154]. Moreover, the presence of NK-T cells was inversely correlated with VEGF ascites levels [155]. Since these cells display the most potent cytotoxicity profile, they might be promising agents for adoptive cell immunotherapy [156]. Further studies are needed to better understand the potential antitumoral capacity of these cells and their role in the different EOC TMEs.

### 3.2. Innate Immune Cells in the Ovarian Cancer TME

#### 3.2.1. NK Cells

Many studies have reported the presence of innate immune cells such as NKs, macrophages and DCs playing important roles in EOC tumorigenesis [103,124,154]. NK cells are crucial effectors in cancer immunosurveillance, recognizing and spontaneously killing virus-infected cells, cancer, and foreign cells hazardous to the host [157]. NK cells mediate antitumoral responses by secreting pro-inflammatory cytokines and chemokines such as IFNγ, TNF, IL-6, GM-CSF and CCL5, which influence antitumor activity and promote innate and adaptive responses in the TME [157,158,159] (Figure 1). Tsiatas et al. analyzed 45 fresh specimens from different EOC and found an increased amount of CD56+ NK and NK-T cells along with activated CD4+ and CD8+ CD25+ T cells in serous and endometrioid carcinomas compared with mucinous and clear cell carcinomas [124]. Despite the high concentration of NKs found in ascites compared to peripheral blood, they are functionally impaired [121,160,161]. The influence of infiltrating NK cells on patient outcome is also debated. Analysis of ovarian carcinoma effusions showed that the presence of NK cells at an advanced stage (IV) predicted worse overall survival [151]. However, a positive antitumoral role for NK cells along with effector CD8+ T cells has been reported [162], and NK cell activity of peripheral blood lymphocytes was related to a significant progression-free survival of EOC patients [163]. Importantly, NK cells are activated or not, according to the balance between inhibitory and activating signals through different NK receptors [157]. Like many other cancers, EOC tumors express NK cell receptor ligand ULBP2, which is an indicator of poor prognosis and could promote T-cell dysfunction in the TME [164] (Figure 2). Since NK cells are important players in antitumoral immunity, more studies aiding to characterize their function, phenotypes, incidence and role in the EOC TME are needed to provide new rational for immunotherapies.

#### 3.2.2. Tumor-Associated Macrophages

Both TAMs and MDSCs constitute up to 20% of the EOC TME and are known to maintain and promote an immunosuppressive TME [103] (Figure 2). TAMs are considered the most abundant infiltrating immune cells in EOC tissue and ascites [165,166]. They possess an immunosuppressive M2 phenotype characterized by the expression of CD163, CD204, CD206, and IL-10 [165], and their presence correlates with tumor progression [140,167]. M2 TAMs secrete colony-stimulating factor 1 (CSF-1) that has been found in high levels in malignant EOC [167], and contributes to tumor growth, invasion, and metastasis. Moreover, EOC cells are able to induce an M2 TAM phenotype [168]. TAMs produce the chemokine CCL22 enabling the trafficking of Tregs to the ovarian tumors [130]. EOC cells as well as TAMs are known to express the coinhibitory molecule B7-H4 [169], a member of the B7 family that has a profound inhibitory effect on the growth, cytokine secretion, and development of T-cell cytotoxicity [169]. B7-H4+ TAMs are able to suppress antitumoral responses in EOC [136]. A study of 103 EOC patients showed that enhanced B7-H4 expression in macrophages correlated with Treg cell numbers in the tumor [136]. Tregs and B7-H4+ TAMs were associated with poor patient outcome. Tregs in the TME can induce B7-H4+ TAMs to produce IL-10 and IL-6 [136], further supporting an immunosuppressive milieu. Higher tumor grade correlated with higher frequencies of CD163+ TAMs [140] and worse progression-free survival [170,171]. Importantly, two studies evaluating M1- (HLA-DR, iNOS) and M2-polarization (CD163, VEGF) markers showed that higher M1/M2 TAMs ratio in tumors was associated with a favorable overall survival [172,173], and high serum levels of CD163 predicts poor EOC patient prognosis [174]. In addition, monocyte-derived macrophages in EOC displayed an altered morphology and defective antitumoral functions including defective antibody-dependent cell-mediated cytotoxicity and phagocytosis [175]. Thus, EOC cells and the TME provoke and maintain a strong immunosuppressive M2 phenotype supportive of tumor progression. Immunotherapeutic approaches aiming to switch TAM phenotypes [176] could help the evolution of antitumoral responses and improve patient outcome.

#### 3.2.3. Myeloid-Derived Suppressor Cells

MDSCs are composed of a heterogeneous population of immature myeloid cells that arise in pathologic conditions such as cancer, inflammation and infection, and possess a potent capacity to dampen T-cell responses [177]. MDSCs are considered key inducers of tumor immune evasion and impaired immunity by upregulating arginase-1, nitric oxide, and reactive oxygen species, and by generating reactive nitrogen species [178] (Figure 2). MDSCs also deplete cysteine, induce Tregs, inhibit T-cell activation and proliferation, attenuate the cytolytic ability of NK cells, and trigger a M2 phenotype [103]. Obermajer et al. showed that the frequencies of CD11b+ CD14+ CD33+ CXCR4+ MDSCs in EOC ascites correlated with CXCL12 and prostaglandin E(2) levels [179]. MDSCs derived from EOC patients also increased gene expression of cancer stem cells, sphere formation and metastasis of EOC [180]. Wu et al. characterized typical monocytic CD14+ HLA-DR^-/lo^ MDSCs in peripheral blood and ascites derived from EOC patients and found that MDSCs are enriched in both compartments [181]. Moreover, the density of MDSCs correlated with poor patient prognosis and elevated levels of IL-6 and IL-10 [181,182]. VEGF expression in EOC induced MDSCs recruitment, inhibiting local immunity [182]. A recent study with mouse EOC cells found that *Snai1*, a major transcription factor that induces epithelial-mesenchymal transition (EMT), mediates EOC progression by upregulating CXCR2 ligands, enabling the recruitment of MDSCs [183]. EOC cells also attracts myeloid cells by producing adenosine [184]. Hence, strategies targeting MDSCs could release the brakes against antitumoral responses. Metformin, a drug used to treat type 2 diabetes, may trigger EOC clinical benefit by improving antitumoral T-cell responses that are impeded by MDSCs in the TME, since this drug can block MDSC suppressor functions by decreasing CD39 and CD73 expression [185].

## 4. Exosomes

Highly proliferating cells such as cancer cells produce large amounts of exosomes which are small (40–100 nm) extracellular vesicles [186]. EOC tumor-derived exosomes carry cell membrane proteins and cargo proteins that could be used for diagnostics (EP-CAM) and immunotherapeutic targeting such as neoantigens and TAAs (Her2-neu, CA-125) [186], proteins (TGF-β1) [187], and miRNAs (miR-21) [188] that are involved in disease progression, metastasis, and chemoresistance [186], as well as immunomodulatory proteins (FAS-L) [189]. Exosomes can be taken up by other cancer cells, CAFs, and immune cells, therefore playing an important role in intercellular communication. Thus far, 2035 exosome cargo molecules have been identified from EOC cells in ExoCarta, a database for exosome cargo [190,191]. Exosomes derived from human patient ascites promotes tumor progression in vivo [189,192], and are proposed to have direct and indirect roles in modulating the immune TME, as exosomes could also be taken up by NKs and B cells [192] (Figure 2). In other disease models, such as melanoma and colorectal cancer, exosomes mediate immunosuppression and immune tolerance by suppressing the activation of T and NK cells, monocytes, modulating T-cell inhibitory molecules expression, and inducing CD8+ T-cell apoptosis [193,194]. FAS-L and TRAIL expression on EOC-derived exosomes inhibit activation of peripheral blood mononuclear cells by DCs through induction of apoptosis [189]. EOC-derived exosomes express ligands (MICA/B and ULBP1-3) for the NK receptor NKG2D, acting as a decoy and interfering with NK-mediated targeting of tumor cells [195]. Greater understanding of the complex network of the intercellular communication between EOC cells, CAFs, and immune cells is needed for the rational design of immunotherapeutic interventions, or leveraged for nanomedicine applications such as TAA loaded-DC-derived exosomes [196] and drug delivery systems [186].

## 5. Cancer-Associated Fibroblasts

CAFs are activated fibroblasts that express α-smooth muscle actin and fibroblast activation protein. They make up 7–85% [197] of the tumor and are the primary stromal cell type in the TME. Cross-talk between epithelial and stromal compartments creates a positive feedback loop, a supportive hyper-activated storm of cytokines, chemokines, angiogenetic factors, and EMT-promoting factors, to promote tumor progression and chemoresistance (Figure 2). CAFs from ovarian cancer patients secrete high levels of hepatocyte growth factor (HGF) that promotes cancer cell proliferation, chemoresistance, invasion, and migration though constitutive activation of cMet/PI3K/Akt pathways and glucose-regulated protein 78 (GRP78) [198,199]. CAFs produce pro-inflammatory cytokines COX-2 and CXCL1 [200], CCL5 [201], CXCL11 [202], and IL-6 [203], which can promote proliferation and EMT. In addition to their direct actions on cancer cells, CAFs also produce exosomes with high levels of TGF-β1 that subsequently activates normal fibroblasts [187]. Interestingly, Givel et al. identified four CAFs subsets in HGSC, finding an accumulation of one subset, CAF-S1, in the mesenchymal molecular subtype of HGSC. CAF-S1 is associated with an immunosuppressive TME, due to its high levels of expression of CXCL12β, which recruits Tregs to the tumor. The CAF-S1 cells also express CD73, B7-H3 and IL-6, which subsequently promote survival and proliferation of Tregs [204]. Thus, CAFs can make major contributions to the creation of an immunosuppressive TME.

On the other hand, EOC cells can stimulate the activation of CAFs by producing high levels of interleukin-1β (IL-1β) [205] and TGF-β [206], which subsequently induces secretion of IL-8, IL-6, IL-1β, VEGF, and growth regulated oncogene-alpha (GRO-α) by CAFs to promote tumor progression [205]. EOC cells release exosomes not only to activate tumor cells, but also to reprogram normal fibroblasts into CAFs [207]. Furthermore, CAFs act on endothelial cells via the secretion of VEGF-C [208] or by upregulating genes such as lipoma-preferred partner, to promote angiogenesis, which leads to tumor progression and chemoresistance [209]. Cross-talk between CAFs and cancer cells, as well as endothelial cells and immune cells, suggests that targeting signaling mechanisms in this relationship may combat chemoresistance and immune modulation better than singly targeting the epithelial compartment.

## 6. Adipocytes and the Omentum

The unique TME of the omentum, a large visceral fat pad that covers the bowel and abdomen cavities [210,211], suggests a two-step model of omental metastasis and tumorigenesis where ovarian cancer cells preferentially and rapidly home to “milky spots” [212] in the omentum, prior to spreading throughout non-“milky spot” areas of the omentum and peritoneal cavity [213,214,215,216,217]. “Milky spots” are highly vascularized regions with aggregates of immune cells, capable of innate and adaptive immune functions, and antigen presentation similar to lymph node structures [212]. The involvement of the omentum and adipose tissue suggests the need to develop intraperitoneal immunotherapy similar to the advances seen with intraperitoneal chemotherapy.

Adipocytes in the omentum produce cytokines and chemokines, including highly secreted IL-6, IL-8, MCP-1, tissue inhibitor of metalloproteinases-1 (TIMP-1) and adiponectin, to promote cancer growth and omental metastases. Adipocytes can alter their lipid metabolism via Fatty acid–binding protein 4 (FAB4) to undergo lipolysis providing fatty acids (FA) to cancer cells as a fuel source for rapid tumor growth [216]. Cancer cells themselves can also alter lipid metabolism, often by upregulating FA receptor CD36 [218] and FAB4 in omental metastases at the tumor/adipocyte interface to promote FA and cholesterol uptake from adipocytes [216] to fuel tumor progression.

Many studies have suggested an association between obesity and the incidence of ovarian cancer as well as an association with poor prognosis [219]. Indeed, in a murine model of ovarian cancer, metastasis and tumor growth is supported in obese mice through altered regulation of FA pathway and increased immunosuppression, demonstrated by a decreased ratio of M1/M2 macrophages [220] (Figure 2). Improved understanding of how adipocytes and the omentum support ovarian cancer growth and promote peritoneal metastases will reveal therapeutic targets for both conventional therapy and immunotherapy. It will be important to consider how age and obesity [221,222,223] may dictate differences in response to immunotherapy and how current models with young, lean mice may fail to accurately model responses to immunotherapy.

## 7. Conclusions

In summary, in order to develop better immunotherapies for EOC we need to identify and consider all key elements found in the TME of not only primary tumors but also in ascites and metastases with a focus on how these features affect and are affected by different cancer therapies. It is crucial to take into account the quality of the TME (immune-activating vs. immune-suppressing mechanisms), tumor immunogenicity, tumor burden mutations, tumor stage, patient overall condition, and age, as well as treatment effects on the TME (chemotherapy, neoadjuvant chemotherapy, surgery debulking). Each of these factors may influence the outcome of EOC and the responses to cancer immunotherapies. Moreover, to avoid tumor recurrence, EOC characteristics such as TAA presentation, expression of coinhibitory molecules, production of immunosuppressive cytokines and chemokines should all be considered to find therapeutic combinations that could synergize and achieve maximal benefits to eliminate EOC. Other articles in this special issue will address some of these topics, including the exploration of promising immunotherapies for HGSC that are currently under investigation [224].

## Figures and Tables

**Table 1:** Human studies using immune checkpoint inhibitors in epithelial ovarian cancers (EOC) (completed or partially completed studies).

Target	Agent	EOC Characteristic	Antitumoral Responses	Immune Related Parameters	Clinical Study
**PD-1**	**Nivolumab** (Opdivo, *BMS-936558, MDX1106*) i.v. infusion every 2 weeks (1 or 3 mg/kg)	Advanced or relapsed platinum-resistant ovarian cancer	A quick antitumor response observed by baseline computed tomographic image, decreased CA-125 blood levels. Overall response: 15%, 2 ^§^ pts had a durable CR, disease control rate in all 20 pts was 45%. Median PFS 3.5 months.	Expression of PD-L1 in ovarian cancer tissues was not significantly correlated with objective response but 16/20 patients having a high expression of PDL1 on tumors did not respond to treatment (vs. 2/4 responders in the PD-L1-low expression group).	Phase II UMIN000005714 [14]
	**Pembrolizumab** (Keytruda, *MK-3475*) i.v. infusion every 2 weeks (10 mg/kg) for up to 2 years	PD-L1+ advanced ovarian cancer	1 * pt CR, 2 pts PR; 6 pts stable disease. Duration of response ≥24 weeks. Overall response was 11.5%. 6/26 (23.1%) had evidence of tumor reduction; 3 had a tumor reduction of at least 30%.	N/A	Phase Ib trial NCT02054806 Active, not recruiting [15]
**PD-L1**	**Avelumab** (Bavencio, *MSB0010718C*) every 2 weeks (10 mg/kg)	Recurrent or refractory ovarian cancer	4/23 (17.4%) pts achieved an unconfirmed best overall response of PR, 11 pts (47.8%) had stable disease, and 2 pts had >30% tumor shrinkage after progression was reported. Median PFS was 11.9 weeks and the PFS rate at 24 weeks was 33.3%.	Exposure to Avelumab significantly increased the ratio of sCD27/sCD40L ^#^. Some antitumor activity of this antibody may be due to ADCC [16].	Phase Ib study NCT01772004 Active, not recruiting [17]
	**BMS-936559** (*MDX-1105*) i.v. infusion every 2 weeks (10 mg/kg) in 6-week cycles	Advanced ovarian cancer	1 of 17 pts (6%) had a PR, and 3 (18%) had stable disease lasting at least 24 weeks.	N/A	Multicenter phase 1 trial NCT00729664 completed [18]
**CTLA-4**	**Ipilimumab** i.v. infusions (10 mg/kg) once every 3 weeks for 4 doses (Induction Phase). Once every 12 weeks (Maintenance Phase), until disease progression or unacceptable toxicity occurs	Recurrent platinum-sensitive ovarian cancer	N/A	N/A	Phase II study (NCT01611558) Active, not recruiting
	**Ipilumimab** Periodic infusions (3 mg/kg) after vaccination with irradiated, autologous tumor cells engineered to secrete GM-CSF (GVAX)	Stage IV ovarian carcinoma	1/9 pts had reduction in circulating CA-125 levels, regression of metastasis, increased humoral reactions to NY-ESO-1. 3 pts achieved stable disease of >6, 4, and 2 months’ duration, as measured by CA-125 levels and radiographic criteria.	The extent of therapy-induced tumor necrosis was linearly related to the natural logarithm of the ratio of intratumoral CD8+ effector T cells to FoxP3+ Tregs in post-treatment biopsies.	[19,20]

^§^ The tumor was histologically identified as clear cell carcinoma in one of the two patients who experienced a CR. * 1 Patient with CR had a PD-L1 gene rearrangement leading to gain of function of the PD-L1 gene secondary to gene amplification, high PD-L1 expression was observed in cancer epithelial cells, as well as high T lymphocyte infiltration (CD4, CD8), some B cells (CD20) and macrophages (CD68) [21]. ^#^ sCD27 is a marker of T-cell activation [22], sCD40L is a measure of immune suppression [23]. Complete response (CR), partial response (PR), patients (pts), progression-free survival (PFS), intravenous (i.v.), antibody-dependent cell-mediated cytotoxicity (ADCC), not available (N/A), Granulocyte-macrophage colony-stimulating factor (GM-CSF), forkhead box P3 (FoxP3), regulatory T cells (Tregs).

**Table 2:** Type and prevalence of tumor-associated antigens (TAAs) in EOC.

TAA Category	TAA	Prevalence (% Patients)	FIGO Stage	References
CTA	OY-TES-1	69% (All subtypes)	I-IV	[24]
	SCP-1	15% (All subtypes)	I-IV	[25]
	SPAG9 ^1^	88% (HGSC)	I-IV	[26]
	AKAP4 ^2^	93% (Serous)	I-IV	[27]
	NY-ESO-1	43% (All Subtypes)	I-IV	[28]
	MAGE-A ^3^	~7–55% (All subtypes)	I-IV	[29,30,31]
Oncogene	p53	Mutation (95% HGSC)/Amplification (35% HGSC)	I-IV	[32,33]
	Her2neu ^4^	35–45% (All subtypes)	I-IV	[34,35,36,37]
	WT1 ^5^	71.4% (LGSC) ~55% (HGSC)	III/IV	[38,39]
	Mesothelin	82% (HGSC)	I-IV	[40,41]
	MUC16 ^6^ (CA-125)	80% (All subtypes)		[42]
Neoantigen	Patient/tumor site specific	Greater number in HR deficient ^7^ tumors	I-IV	[12,43]

^1^ SPAG9: Sperm-associated antigen 9. ^2^ AKAP4: A-kinase anchoring protein 4. ^3^ MAGE-A: Melanoma antigen. ^4^ Her2-neu: human epidermal growth factor receptor 2-neu. ^5^ WT1: Wilms’ tumor 1. ^6^ MUC16: Mucin-16. ^7^ *BRCA1/BRCA2*, Fanconi anemia genes (*PALB2*, *FANCA*, *FANCI*, *FANCL*, and *FANCC*), restriction site associated DNA genes (*RAD50*, *RAD51*, *RAD51C*, and *RAD54L*), DNA damage response genes (*ATM*, *ATR*, *CHEK1*, and *CHEK2*). High-grade serous ovarian cancer (HGSC), low-grade serous ovarian cancer (LGSC).

**Table 3:** TAA targeted immunotherapies in EOC.

TAA Category	TAA	Immunotherapy	References
CTA	NY-ESO-1	Recombinant protein vaccine (Epitope ESO_157–170_) + Incomplete Freund’s Adjuvant	[44]
		Overlapping long peptides + Montanide/Poly-ICLC adjuvants	[45]
		NY-ESO-1b + Montanide	[46]
		Recombinant vaccinia prime-NY-ESO-1 (rV-NY-ESO-1) + recombinant fowlpox boost-NY-ESO-	[47]
		1 (rF-NY-ESO-1)	
		NY-ESO-1-specific engineered T Cells	(NCT03159585, NCT03017131, NCT02457650)
		NYESO-1(C259) transduced autologous T cells	(NCT01567891)
	MAGE-A	Autologous genetically modified MAGE-A4^c^¹º³²T cells	(NCT03132922)
Oncogene	p53	Modified vaccinia Ankara vaccine vs. wild-type human p53 (p53MVA) + gemcitabine	[48]
		Synthetic long peptide (SLP) vaccine	[49]
	Her2neu	Her2-neu peptide vaccine	(NCT00194714)
		Exvivo Her2-neu specific T-cell expansion	(NCT00228358)
	WT1	Autologous WT1 T Cells + Cyclophosphamide + Fludarabine	(NCT00562640)
		WT1 peptide vaccine + Montanide + GM-CSF + Nivolumab (PD-1)	(NCT02737787)
		WT1 mRNA-loaded DCs^2^	[50]
		WT1 peptide vaccine + Montanide	[51]
	Mesothelin	Anti-Mesothelin CAR-T ^1^ cells	[41]/(NCT02580747)
	MUC16 (CA-125)	Antibody therapy (Oregovomab, ACA125/Abagovomab)	[52,53,54,55]
		CAR-T Therapy + IL-12	[56,57] (NCT02498912)
Neoantigen	Patient/tumor site specific	Autologous DCs pulsed with oxidized autologous whole-tumor cell lysate + bevacizumab + cyclophosphamide	[58]
		Autologous neoantigen engineered T-Cells	(NCT03412877)

^1^ Chimeric antigen receptor T cell (CAR-T). ^2^ Dendritic cells (DCs).
